# The physical and psychological problems of immigrants to Japan who require psychosomatic care: a retrospective observation study

**DOI:** 10.1186/s13030-016-0052-x

**Published:** 2016-02-24

**Authors:** Atsuko Koyama, Hirokuni Okumi, Hiromichi Matsuoka, Chihiro Makimura, Ryo Sakamoto, Kiyohiro Sakai

**Affiliations:** Department of Psychosomatic Medicine, Kinki University, Faculty of Medicine, 377-2, Ohno-higashi, Osaskasayama City, Osaka 589-8511 Japan

**Keywords:** Immigrants, Psychosomatic medicine, Cultural differences, Language barriers, Psychological distress, Medical interpreter

## Abstract

**Background:**

As the number of immigrants to Japan increases, the health problems of foreign nationals also have an increasing impact on Japanese medical institutions. The aim of this study was to clarify the Japan–specific health problems related to both the physical and psychological symptoms of foreign nationals from the viewpoint of psychosomatic medicine. The second aim was to clarify the measures that should be taken in Japan and similar countries where immigration may still be considered less than common.

**Case Presentation:**

The study period was from June 2004 to May 2015. The data of non-Japanese patients who had visited the Department of Psychosomatic Medicine, Kinki University Hospital and its branches, Sakai Hospital and Nihonbashi Clinic, were collected. All patients were aged 16 years or over. Multiple factors, such as age, sex, nationality, length of stay, marital status, employment status, level of Japanese proficiency, clinical symptoms, physical and psychiatric diagnosis, psycho-social factors and therapy were retrospectively analyzed from the medical charts of 20 non-Japanese patients. Cases were divided into two groups; early onset and late onset cases. This study showed that multiple factors related to the health problems of non-Japanese patients were combined and had a mutual influence, however, they can be summarized into two important clinical observations. These are 1) cultural differences, and 2) language barriers related to both the physical and psychological symptoms of non-Japanese patients from the viewpoint of psychosomatic medicine.

**Conclusions:**

Future efforts should focus on sensitizing health care professionals in Japan to the psychosomatic problems of non-Japanese patients as well as on facilitating medical systems with services such as medical professional interpreters and liaison-consultation models. It is essential to take measures against language barriers and to promote the field of transcultural psychiatry and psychosomatic medicine in Japan. In addition, the Japanese government should introduce a more comprehensive social support system for non-Japanese people.

## Background

Due to the increase in international migration all over the world, the health problems of migrants have also been rising. Several reports of physical and mental health characteristics have been published in the US, Canada, Australia, and Europe. These papers clarified the multiple factors related to the health problems of immigrants, such as the effects of social connections (including family connections, relative support, friend support, and neighborhood cohesion), socioeconomic status and immigration-related factors (including nativity, length of residence in the country, and language proficiency) [1].

**Fig. 1:**
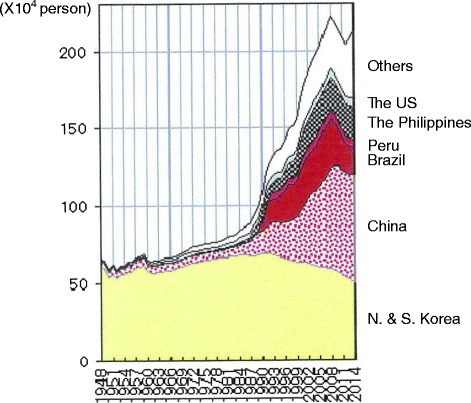
The change in the number of immigrants to Japan. Data acquired by the Ministry of Justice (Japan). The total number of immigrants has been gradually increasing, with a particularly sharp increase since 1990. After the Fukushima earthquake and consequent nuclear disaster in 2011, the number of immigrants decreased in response. China includes Hong Kong and Taiwan for the purpose of this report.

Unlike western countries with more liberal immigration policies, Japan has been quite adverse to accepting foreign nationals, including asylum seekers [2]. The foreign population of Japan was just under 2% in 2014 (foreign nationals living in Japan / total population = 2,121,831 / 127,083,000 = 1.67%) [3], and strict visa rules are in place regarding work and marriage. Reasons for such adversity include Japan being overpopulated for its size, as well as the belief that outsiders may disrupt Japanese society due to it being an island nation. Nevertheless, the rate of immigration has been increasing year by year [4] (Fig. 1). Furthermore, with the continued decrease in Japan’s birthrate and increase in the number of elderly citizens, there exists a high possibility that immigration will be increased in order to maintain financial stability. Accordingly, the health problems of foreign nationals are having an increased impact on Japanese medicine. However, Japan’s hospitals have had less preparation for an influx of foreign patients than other countries because of past restrictions on accepting immigrants. Consequently, Japan is unfamiliar with the health problems of immigrants, and it is unknown whether there are problems specific to Japan or not. A field of medicine that some foreign nationals may have had contact with is psychosomatic medicine. Psychosomatic medicine doctors deal with both stress-related physical symptoms and psychological distress. Because of this, psychosomatic medicine doctors in Japan are more likely to attend to the psychological distress of foreign nationals than doctors of other departments.

The aim of this study was to clarify the Japan–specific health problems of non-Japanese patients related to both physical and psychological symptoms from the viewpoint of psychosomatic medicine. The second aim was to clarify the measures that should be taken in Japan and similar countries where immigration may still be considered less than common.

## Case Presentation

### Methods

#### Patients

The inclusion criteria of the patients in this study were as follows:

1. Foreign nationals who had visited the Department of Psychosomatic Medicine, Kinki University Hospital and its branches, Sakai Hospital and Nihonbashi Clinic from June 2004 to May 2015.
2. Patients who had physical symptoms due to psychosocial distress
3. Those aged 16 years or over

The exclusion criteria were as follows:

1. Patients with primary psychiatric diseases, for example having hallucination and/or delusion
2. Foreign nationals visiting Japan on tourist visas
3. Patients who were unwilling to participate in this study and contacted us to refuse participation

### Design and setting

This was planned as a case series study.

All patients who visited our department for the first time filled out a systemic medical questionnaire including their demographic background, subjective physical complaints, and psychological distress, after which semi-structured interviews were performed by doctors. The distress was classified into four factors; physical, psychological, social distress, and spiritual pain; as is done for the “total pain” of cancer patients.

During the study period, all items assessed during routine clinical practice were extracted from the patients’ medical charts, including age, sex, nationality, length of stay, marital status, employment status, clinical symptoms, physical and psychiatric diagnosis, psycho-social factors and therapy. In order to make an objective assessment, multiple independent researchers, using the same standards, analyzed and discussed the psycho-social factors of each case.

The level of Japanese proficiency was evaluated and separated into four levels: Level 1. Unable to communicate in Japanese at all; Level 2. Able to exchange greetings but not express thoughts sufficiently; Level 3. Able to understand and participate in daily conversation but still having limitations when communicating; and Level 4. Able to communicate sufficiently in Japanese.

The Self-rating Depression Scale (SDS) [5] and the State-Trait Anxiety Inventory (STAI) [6] were used to evaluate emotional distress in terms of depression and anxiety. In SDS, a cut-off score of 50 was adopted for depressive state. In STAI, cut-off scores of 42/45 (STAI-S/T for women) and 41/44 (STAI-S/T for men) were adopted for the assessment of the tendency toward anxiety.

Physical diseases such as irritable bowel syndrome, headache, and asthma were diagnosed as psychosomatic disorders based on “A guideline for the diagnosis and treatment of psychosomatic disorders 2006” [7]. Each patient’s mental status was evaluated via a formal medical interview, leading to a diagnosis based on the Diagnostic and Statistical Manual of Mental Disorders, Fourth Edition, Text Revision (DSM-IV-TR) [8].

## Results

### Demographic and clinical characteristics

Approximately 2,900 new patients come to Kinki University Hospital and its branches, Sakai Hospital and Nihonbashi Clinic, and 20 to 25 new patients visit the Department of Psychosomatic Medicine every month. Although the exact number of non-Japanese patients is unclear, twenty cases were eligible for this study according to the inclusion criteria.

Detailed demographic characteristics of the patients are listed in Table 1. There were ten male and ten female patients. The age of patients ranged from 23 to 66, with an average age of 39.2 ± 11.5 years. Nine patients were native English speakers from the USA, England, Australia or New Zealand and eleven were of Asian origin, from China, Taiwan, Korea, Indonesia, or Mongolia. Their length of stay in Japan at the time of their first visit to our department ranged from one month to 30 years. Seven were language teachers, one was a student, and eight were unemployed. Their Japanese proficiency ranged from level 1 to 4, and all patients except for case 15, had some degree of language limitation that hindered communication with the medical staff. History taking, physical examination, explanation of results, and supportive sessions were conducted in English for the eleven patients fluent in English (cases 1–9, 19, 20), and in Japanese for the others. Case 11 brought a Chinese-Japanese interpreter.

**Table 1:** Profiles of patients

No	Age/Sex	Nationality	Length of stay	Marital status	Employment status	Japanese proficiency	SDS	STAI S/T	Physical symptoms	Physical (Psychosomatic) diagnosis	Psycho-logical symptoms	Psychiatric diagnosis	Psycho-social factors	Therapy
1	23/M	USA	1m	Single	English teacher	2	35	-	Cataplectic attack	-	Anxiety	AD w/anxiety	Culture shock	Anxyolytics SSRI
2	23/F	USA	2m	Single	English teacher	1	57	-	Epigastralgia Nausea	Functional dyspepsia	Anxiety	AD Mixed type	Culture shock	Anxyolytics
3	66/F	USA	28y	Divorced	Unemployed	3	48	54/56	Palpitation Dizziness	-	Insomnia Anxiety	Anxiety disorder	Divorce Dismissed from job	Hypnotics
4	28/F	USA	3y	Married	Unemployed	2	54	55/51	Appetite loss	-	Insomnia Depressive mood	postnatal MDD	Isolation Cultural differences	Antidepressants
5	27/F	USA	3y	Single	English teacher	2	29	47/41	Eruption Body itching	Urticaria	Anxiety	AD w/anxiety	Job-related stress	Anxyolytics Anti-allergic drug
6	41/M	England	7y	Married	English teacher (self employed)	3	49	35/41	Palpitation Sweating	-	Anxiety	Panic disorder	Conflict w/spouse job-related stress	Antidepressants (SSRI)
7	58/F	Australia	30y	Bereaved	English teacher	3	57	53/47	Headache Back pain	Chronic pain syndrome	Anxiety Depressive mood	AD Mixed type	Death of husband	Anxyolytics
8	40/M	Australia	4y	Married	Unemployed	2	58	50/54	Fatigue Headache	Tension Headache	Insomnia Depressive mood	MDD	Dismissed from job	Antidepressants Anxyolytics
9	38/M	New Zealand	10y	Single	English teacher	2	42	44/53	Dyspnea Chest pain	Asthma	Depressive mood	Acute stress disorder	Breakup w/partner	Anxyolytics
10	56/F	China	14y	Married	Unemployed	2	-	-	Fatigue Diarrhea	Irritable bowel syndrome	Apathy Depressive mood	MDD	Dismissed from jpb Conflict w/spouse	Hospitalization Antidepressants
11	42/M	China	10y	Married	Unemployed	1	-	-	Fatigue Dizziness	Unstable Hypertension	Insomnia	AD Mixed type	Dismissed from job	Hypnotics Herbal medicine
12	51/M	China	14y	Married	Unemployed	2	46	52/40	Abdominal pain	Chronic pain syndrome	Anxiety	Anxiety disorder	Language barriers	Anxyolytics
13	36/F	China	5y	Married	Unemployed	2	47	56/48	Epigastralgia Dizziness	Hepatitis type B Acute exacerbation	Anxiety	Anxiety disorder	Language barriers	Hospitalization
14	39/F	China	8y	Single	Employee	2	42	57/55	Sweating Tremors	-	Anxiety	Anxiety disorder	Language barriers	Anxyolytics
15	37/F	Taiwan	19y	Single	Chinese teacher	4	58	57/50	Epigastralgia Appetite loss	Functional dyspepsia	Insomnia	AD Mixed type	Breakup w/ partner	Hospitalization Anxyolytics
16	22/F	Taiwan	3y	Single	Student	3	47	53/51	Subfever Fatigue	-	Suicidal	MDD	Maladaptation to school life	Rest Counseling
17	38/M	Korea	12y	Married	Employee	4	48	50/52	Headache Dizziness	Tension Headache	Insomnia Depressive mood	AD Mixed type	Job-related stress	Anxyolytics Antidepressants AT
18	42/M	Korea	10y	Married	Priest	4	55	48/42	Chronic pain Dizziness	Chronic pain syndrome	Insomnia Depressive mood	MDD	Traffic accident Job-related stress	Antidepressants Hypnotics Counseling
19	40/M	Indonesia	12y	Divorced	Self-employed	2	44	52/50	Chest pain Palpitation	Chronic pain syndrome	Anxiety	AD w/anxiety	Job-related stress	Anxyolytics AT
20	37/M	Mongolia	7y	Married	Unemployed	3	28	25/29	Facial eruptions	<Lung cancer>	Anxiety	AD Unspecified	<Lung cancer>	Anxyolytics

*M* Male, *F* Female, *m* month, *y* year, *AD* Adjustment disorder, *MDD* Major depressive disorder, *SSRI* Selective Serotonin Reuptake Inhibitor, *AT* Autogenic training

The level of Japanese proficiency

Level 1. Unable to communicate in Japanese at all

Level 2. Able to exchange greetings but not express thoughts sufficiently

Level 3. Able to understand and participate in daily conversation but still having limitations when communicating

Level 4. Able to communicate sufficiently in Japanese

Clinical characteristics are listed in Table 1. Because our department is classified as internal medicine and attends to patients complaining of physical symptoms due to psychosocial distress, the patients had various types of physical symptoms, mainly general fatigue, abdominal pain/epigastralgia, and palpitation. The main psychological symptoms were anxiety, depressive mood, and insomnia.

The physical (psychosomatic) disease and psychiatric diagnoses are shown in Table 1. Cases 10 and 11 did not complete the SDS and STAI due to a lack of literacy in English or Japanese. Cases 1 and 2 neglected to complete their STAI forms. The average SDS scores of the remaining eighteen patients were 46.9 ± 9.0, with six patients having higher scores than the cut-off of 50. Among the sixteen patients who completed STAI-S/T, seven female patients (cases 3, 4, 7, 13–16) had higher scores than the cut-off of 42/45 for females and four male patients (cases 8, 9, 17, 19) had higher scores than the cut-off of 41/44 for males. Nine cases (1, 2, 5, 7, 11, 15, 17, 19, 20) were diagnosed as having adjustment disorders, and subtypes were determined by referencing the SDS/STAI scores and patients’ symptoms. Five cases (4, 8, 10, 16, 18) were diagnosed as having major depressive disorder, four cases (3, 12–14) with anxiety disorders, and the remaining cases with stress disorder and panic disorder. Three of the twenty patients needed to be hospitalized for a short period.

The psycho-social factors ranged widely, from culture shock, maladjustment to Japanese society, break-up or conflict with a partner and/or family member(s), and work-related social problems to serious physical diseases such as lung cancer and acute exacerbation of hepatitis type B. The basic underlying problems were cultural differences and communication difficulties between these non-Japanese patients and their Japanese partners, family members, co-workers, and medical staff due to the language barrier.

### Case details

#### Early onset cases

Having studied Japanese and Karate prior to his arrival in Japan, the patient in case 1 had had various expectations of his life in Japan. However, the reality was different from what he had imagined. He suffered a cataplectic attack and was diagnosed with adjustment disorder and possibly panic disorder.

The patient in case 2 was an assistant English language teacher at a junior high school. She had become frustrated and felt alienated due to a lack of conversation with her co-workers and because she was much younger than them. She felt the atmosphere in the teachers’ room was unfriendly. On her working days, she developed a sense of isolation and suffered from epigastralgia and nausea.

### *Late onset cases*

The patient in case 3 had been in Japan for more than 20 years and had been working as a social worker and English teacher at a private conversation school. She was dismissed from the English school due to a lack of students. She was divorced from her Japanese husband and had three grown-up children who lived apart from her. She suffered insomnia and anxiety about her future. Despite having friends and being a member of the local church, she felt a sense of solitude living in Japan.

The patient in case 4 complained of appetite loss, insomnia, and depressive mood since giving birth. She did not have any support from her Japanese husband’s family members or community support, and she was diagnosed with postnatal major depression.

The patient in case 5 was a single 27 year-old English teacher. When she had job-related stress such as conflict with co-workers due to differences in work approach, she manifested urticaria and anxiety.

The patient in case 6, a 41 year-old man ran an English conversation school with his wife. When he had job-related stress and conflicts with his wife, he felt a sense of isolation and had palpitations and sweating attacks. According to the criteria of DSM-IV-R, he was diagnosed with panic disorder, and an antidepressant, a Selective Serotonin Reuptake Inhibitor (SSRI), was effective.

The patient in case 7 was shocked by her husband’s sudden death and fell into a depressive mood. Although she had been in Japan for 30 years and her Japanese proficiency level was 3 according to our scale, she had not yet established any close human relationships with Japanese members of the community. She was concerned about her future and complained of various types of physical pain, such as headache and back pain.

After the patient in case 8 lost his job, his wife worked fulltime and he became a ‘house husband’, doing housework and taking care of their children, in what is usually regarded as a female role, particularly in Asian societies. He complained of fatigue and headache and gradually became depressive.

The patient in case 9 complained of dyspnea and chest pain, and was diagnosed with asthma. His physical symptoms occurred just after breaking up with his girlfriend, so his state was considered to be acute stress disorder. Anxyolytics somewhat mitigated both his physical and psychological symptoms.

The patient in case 10 was a 56 year old Chinese man. He had been in Japan for 14 years and was married to a Chinese national. After losing his job, it was difficult for him to find a new one and he remained unemployed for three years. He suffered from irritable bowel syndrome and gradually fell into depression. When his wife sometimes complained about his unemployment, his depressive state was exacerbated and he needed hospitalization.

The patient in case 11 had been working for an automobile factory. He had artificial hip joint replacement surgery and had to resign from his job. Because his Japanese proficiency level is very low (Level 1 on our scale), he could not find a new job and had to live on welfare. He had been suffering from both depressive mood and anxiety, and complained of various types of physical symptoms, such as fatigue, dizziness, and unstable blood pressure.

The patient in case 12 had had several operations for abdominal pain. In the first operation, he suffered from subileus and needed to have an operation to improve it. Months later, he again complained of abdominal pain and was referred to an emergency hospital. Because he could not explain his symptoms precisely in Japanese, the doctors had to refer to his past history of subileus, and the same operation was again performed on him. The same situation repeated itself several times and consequently the patient developed a strong anticipation anxiety toward abdominal pain.

The patient in case 13 complained of epigastralgia and dizziness, but could not explain her past history of hepatitis type B sufficiently in Japanese and her doctor could not immediately make a differential diagnosis.

The patient in case 14 also suffered from sweating and tremors when she felt anxiety about her medical condition and her future, but she could not explain her feelings appropriately at the hospital.

The patient in case 15, a single Taiwanese woman, had been in Japan for 19 years and had a steady job as a Chinese teacher. She manifested epigastralgia and appetite loss and was diagnosed with functional dyspepsia just after breaking up with her partner.

The patient in case 16 was a student at a vocational school and reported suicidal ideation because she could not make friends and felt isolated.

The patient in case 17 was a 38 year old, married Korean employee. After he was promoted in a Japanese company, he had a human relationship issue with his Japanese subordinates and complained of headache and dizziness and was diagnosed with adjustment disorder with mixed types. The combination of medication with anxyolytics and antidepressants and autogenic training for relaxation was effective.

The patient in case 18 was a Christian priest. He had had difficulties in the past with missionary work and psychological distress because Japan lacks a strong background in Christianity and, furthermore, he developed chronic pain syndrome and dizziness in the wake of a traffic accident.

The patient in case 19 was a 40 year old, self-employed Indonesian. He was under pressure at his company because the management system in Japan is different from his home country. He suffered from adjustment disorder with anxiety and complained of chest pain and palpitation.

The patient in case 20 complained of anxiety related to his lung cancer diagnosis. He was a devout Buddhist and always contemplating the meaning of life, which is commonly connected to the spiritual pain of cancer patients.

## Discussion

This study showed that multiple factors related to the health problems of non-Japanese patients combined and had a mutual influence on the disease of these patients, however, they can be summarized into two important clinical observations. There are cultural differences and language barriers related to both the physical and psychological symptoms of non-Japanese patients that require intervention in the form of psychosomatic medicine.

### Cultural differences

The length of stay in Japan of our cases was between one month and 30 years. This time frame may be related to the heterogeneity of the cases. Thus, early onset cases (cases 1, 2) and late onset cases (cases 3–20) should be discussed separately. In addition, careful attention must be given to the wide range of cultural differences the patients will exhibit.

### Culture shock due to change of environment

Early onset cases (cases 1, 2) were the result of maladaptation to a change of environment and culture shock [9]. This type of reaction could occur in every immigrant. However, Asian culture is very different from Western culture, so two American patients suffered culture shock and could not adjust themselves to Japanese society. Recently, the Japanese government has promoted the use of native English-speaking teachers as assistant language teachers with the Japan Exchange and Teaching Programme [10]. In proportion to this increase in numbers, these types of early onset cases might also increase. Accordingly support and prevention measures should be introduced.

### Lack of social connections

Groups from Western societies tend to initially come to Japan alone for short-term experiences. Some of them find a partner in Japan, marry, and continue to live in Japan for a long time. Conflict or breaking up with a partner is one of the most stressful life events for people [11]. An additional aspect foreign nationals living in Japan need to cope with is a gap in values with their Japanese partners. For example, in Japanese society, raising children is the primary concern for almost all married couples, however, in Western society the relationship between wife and husband is the main priority in family life. This difference sometimes brings conflict to couples made up of non-Japanese and Japanese people. Moreover, the relationship between a husband/wife and their relatives-in-law of a different nationality can be more difficult than relationships with those of the same nationality, due to cultural differences. In addition, if their relatives live in foreign countries, couples may not receive enough support when raising children. Lack of support from relatives, friends, or the community is a serious problem, and mixed nationality couples tend to be isolated. This factor has limited influence on couples that maintain a good relationship. However, patients who have suffered a relationship break up or conflict may suffer psychological damage due to a lack of support. Such situations could be seen in cases 3, 4, 6, 7, 9, 10, and 15. These patients seemed to adjust to Japanese society, however, once problems occurred, their fragile social environments may have been factors that exacerbated their symptoms.

On the contrary, groups from East Asia tend to immigrate to Japan with their family members for economic reasons. They often live in a community made up of people from the same country. Though it can be supportive to newcomers, it sometimes hinders them from integrating into Japanese society because they do not have enough chances to communicate with Japanese residents or co-workers outside of their community or family. The patient in case 16 suffered from maladaptation to school life due to a lack of support from her peers, illustrating another example of the importance of communication support.

More comprehensive social support for non-Japanese people needs to be introduced by the Japanese government because the continuing increase in immigrants is inevitable due to necessary measures being taken to combat the declining birthrate and rapid aging of Japanese society.

### Work problems

In the workforce, once non-Japanese people become unemployed, finding new employment can be difficult [12], as seen in cases 3, 8 and 11. One reason for this is that the Japanese government restricts the hiring of foreign workers to professional or technical fields. Generally speaking, groups from Western societies often come to Japan as a language teacher or an executive technician, so once they are dismissed from a job it could be difficult for them to find a new job with the same working conditions, as in cases 3 and 8. Due to the conditions of their visas, they may also be restricted from working in other areas. On the other hand, groups from East Asia often come to Japan for economic reasons, with them perceiving Japan as having more opportunities than their homeland. Most of them find work as blue-collar workers and, once they lose a job due to disease (case 10) or injury (case 11), it is difficult for them to find a new job and to maintain their economic level. Some of them need welfare support from the Japanese government, as seen in case 10.

Alternatively, the patients in cases 5, 6, and 17–19 had job-related stress due to differences in attitude toward the work at hand. Traditional Japanese working culture, such as lifetime employment and a seniority wage system, continues to survive in some companies and may be alien to those unfamiliar with such systems. Similarly, the philosophy that work is a higher priority than personal commitments sometimes conflicts with Western values. Non-Japanese patients experienced trouble with their co-workers and partners stemming from such differences in values. In addition, non-Japanese patients could not communicate competently with people around them due to the language barrier. Thus, in many cases cultural differences and the language barrier can combine to further complicate the situation.

### Religious issues

Another manifestation of cultural differences concerns religious issues, which were revealed in cases 18 and 20. In general, despite the majority being Buddhist from birth, Japanese people lack a strong religious background and, due to this lack of understanding, we could not give sufficient psychological support to some non-Japanese patients.

### Language barrier

The second significant factor is the language barrier. All patients in our study had experienced language based communication problems, regardless of their length of stay in Japan, which ranged from 1 month to 30 years. Difficulties in communication with medical staff can lead to serious misdiagnosis and bring grave anxiety to patients, as seen in cases 12–14.

There were some limitations in psychotherapy due to the language barrier and, as a result, we could not give sufficient medical care to these patients. Without an interpreter non-Japanese patients were unable to clearly express their psychological issues. Among health problems, psychological distress is especially difficult to express in a foreign language, anywhere in the world [13, 14]. Even in English speaking countries that have more experience with immigration, such as Canada and Australia, immigrants are reluctant to use mental health services due to the language barrier [15–18]. Though a number of Japanese doctors are fluent in English, which enables them to communicate freely with patients with an English speaking background, patients who speak languages other than Japanese and English have trouble expressing themselves clearly and being understood. Nationals from Japan’s immediate neighbors China and Korea make up the main immigrant groups in Japan, and many are unable to speak either Japanese or English. This failure of communication sometimes brings about serious results, such as the misdiagnosis in case 12. Therefore, the language barrier here does not consist of only English proficiency, as in the US, but of multiple languages. In many situations, patients bring their relatives or friends as an ad hoc interpreter, for example the patient in case 8 brought his wife. However, professional medical interpreters are more effective than ad hoc interpreters [19] and a basic system of medical interpreters [20], including remote-simultaneous interpretation [21], should be established. Medical interpreters of various languages have already been introduced to several hospitals in Japan, but their number is still insufficient. Firm educational and occupational systems are needed. Moreover, a specified medical service similar to what our department provides, in which medical staff communicate with patients in English as a mutual communication tool, should be expanded. Currently, 48 psychiatrists/counselors are certified by the Japanese Society of Transcultural Psychiatry as transcultural specified advisers who deal with the mental health of non-Japanese patients/clients [22], however this number is too small. It is essential to take measures against language barriers and to promote the fields of transcultural psychiatry [23] and psychosomatic medicine [24–26] in Japan.

### The role of psychosomatic medicine

Psychosomatic medicine was established in Japan in 1996 as one of the specific medical fields in which “psychosomatic disorders” are dealt with, and it has been developing widely in both Japan and Germany. Psychosomatic disorders are defined as physical diseases whose onset and course are closely related to psychosocial factors and contain both organic and functional disorders [27]. The department of psychosomatic medicine is classified as part of internal medicine, not psychiatry, and attends to patients complaining of physical symptoms due to psychosocial distress. Therefore, one of the exclusion criteria of this study was patients with primary psychiatric diseases, for example having hallucination and/or delusion, and it is understandable that not a single case has been diagnosed with a posttraumatic stress-related disorder. The types of patients’ stress varied from culture shock to breaking up with a partner and dismissal from employment. When patients came to our department complaining of only physical symptoms, their psychosocial distress should be taken into consideration in order to make an appropriate psychological intervention. In this way, psychosomatic medicine doctors should pay more attention to non-Japanese patients’ cultural backgrounds and play an important role in supporting the growing number of immigrants to Japan.

### Limitations

The present study has several limitations. First, this study was based on consultation cases only in our hospital. Further studies are needed to investigate the present treatment strategies for non-Japanese patients all over Japan and to assess the significance of our practice. In addition, our hospital is located in Osaka, which is the third biggest city in Japan and is home to many foreign nationals of Asian origin. There were no patients in this study from the Philippines, Peru, or Brazil, who make up approximately 25% of the total immigrants to Japan (Fig. 1). Second, the length of stay in Japan in our cases was between one month and 30 years, so this time frame might be partially responsible for the heterogeneity of the cases. Therefore, early onset cases (cases 1,2) and late onset cases (cases 3–20) were discussed separately. However, there was some common background from the viewpoint of cultural differences. Third, this study is spread over a long period, twelve years, so the circumstances of immigrants have changed drastically in Japan, however, the essential issues have continued, and we were able to recognize important, universal points through the examination of our cases. At present, globalization has been spreading rapidly and new issues are arising, such as asylum seekers and medical tourism, so the health problems of non-natives is becoming more important.

Although our study has several limitations, some highly suggestive results were seen as helpful information for clinical psychosomatic practice for non-Japanese patients and for suggesting future studies. In order to elucidate the significance of a specific outpatient service for non-Japanese patients and to promote psychosomatic medicine, further research addressing the present study’s limitations is necessary.

## Conclusions

In conclusion, this study focused on the physical and psychological health problems of foreign nationals who visited the department of psychosomatic medicine. It provides preliminary findings of the characteristics of the psycho-social factors affecting non-Japanese patients. The basic underlying problems were cultural differences and communication difficulties due to a language barrier. Non-Japanese patients complained of various types of psychological and physical symptoms, therefore future efforts should focus on sensitizing health care professionals from all fields, not only psychiatrists, in Japan to the psychosomatic problems of expatriates. In addition, it is necessary to facilitate medical systems with services such as medical professional interpreters and liaison-consultation models. The Japanese government should introduce a more comprehensive social support system for non-Japanese people, as a continued increase in immigration is inevitable due to necessary measures implemented to combat the declining birthrate and rapidly aging Japanese society.

Although the health problems of immigrants have been well studied in the US, Canada, Australia, and Europe, we believe that our study has important implications for countries, particularly in Asia, where the number of immigrants has been increasing due to the spread of globalization.

### Consent

This study was approved by the ethics committee of our hospital (No. 27–004). We paid attention not to infringe on patient privacy as much as possible and publicly displayed information regarding this study on the homepage of our department (http://www.kindai-psychosomatics.com/) so that those unwilling to participate could contact us to refuse participation.

## Abbreviations

SDS: Self-rating Depression Scale
STAI: State-Trait Anxiety Inventory
SSRI: Selective Serotonin Reuptake Inhibitor
